# AKI in Severe Legionnaires’ Disease in Intensive Care Unit

**DOI:** 10.1016/j.ekir.2026.107018

**Published:** 2026-08-12

**Authors:** Mickaël Bobot, Emile Charruyer, Romain Arrestier, Noémie Resseguier, Gael Piton, Jean-Christophe Richard, Laurent Argaud, François Arrive, Sami Hraiech, Jean-Pierre Quenot, Guillaume Louis, Damien Barrau, Nacim Benchabane, Nicolas Bruder, Come Bureau, Julien Carvelli, Damien Contou, Cédric Daubin, Nina De Montmollin, Pierre-François Dequin, Julien Dessajan, Paul Gabarre, Emmanuel Guerot, Pauline Guillot, Camille Haumont, Hervé Hyvernat, Mathieu Jamme, Laurent Lefebvre, Julien Moury, Jonathan Nicolas, Saad Nseir, Noémie Peres, Jean-Damien Ricard, Benjamine Sarton, Luca Servan, Pierre Simeone, Bertrand Souweine, Lara Zafrani, Benjamin Zuber, Paul Chanareille, Julien Demiselle, Guillaume Voiriot, The LegionellAKI Study Group, Laurent Argaud, Romain Arrestier, François Arrive, Damien Barrau, Nacim Benchabane, Mickaël Bobot, Nicolas Bruder, Come Bureau, Julien Carvelli, Emile Charruyer, Khalil Chaibi, Paul Chanareille, Jonathan Chelly, Damien Contou, Cédric Daubin, Nina De Montmollin, Julien Demiselle, Pierre-François Dequin, Julien Dessajan, Paul Dunand, Alexandre Elabbadi, Julien Faraut, Paul Gabarre, Emmanuel Guerot, Pauline Guillot, Camille Haumont, Sami Hraiech, Hervé Hyvernat, Mathieu Jamme, Kada Klouche, Laurent Lefebvre, Marc Leone, Guillaume Louis, Julien Maizel, Julien Moury, Jonathan Nicolas, Saad Nseir, Benoit Painvain, Noémie Peres, Gaël Piton, Jean-Pierre Quenot, Noémie Resseguier, Jean-Damien Ricard, Jean-Christophe Richard, Benjamine Sarton, Luca Servan, Pierre Simeone, Bertrand Souweine, Guillaume Voiriot, Lara Zafrani, Benjamin Zuber

**Affiliations:** 1 Centre de Néphrologie et Transplantation Rénale, Hôpital de la Conception, AP-HM, Marseille, France; 2 Université d'Aix-Marseille, INSERM 1263, INRAE 1260, C2VN, Marseille, France; 3 Assistance Publique – Hôpitaux de Paris, Hôpital Tenon, DMU APPROCHES, Service de Médecine Intensive Réanimation, Paris, France; 4 Service de Médecine Intensive Réanimation Médicale, Hôpital Mondor, AP-HP, Créteil, France; 5 Laboratoire de Santé Publique, CERESS, Université d'Aix-Marseille, Marseille, France; 6 Réanimation Médicale, CHU de Besançon, Besançon, France; 7 Service de Médecine Intensive Réanimation, Hôpital de la Croix Rousse, HCL, Lyon, France; 8 Service de Médecine Intensive Réanimation, Hôpital Edouard Herriot, HCL, Lyon, France; 9 Service de Médecine Intensive Réanimation, CHU de Poitiers, Poitiers, France; 10 Assistance Publique - Hôpitaux de Marseille, Hôpital Nord, Médecine Intensive Réanimation, Centre d'Etudes et de Recherches sur les Services de Santé et qualité de vie EA 3279, Aix-Marseille Univ, Marseille, France; 11 Université Bourgogne-Europe, CHU Dijon-Bourgogne, Service de Médecine Intensive-Réanimation, INSERM CIC-1432-Equipe Lipness UMR 1231, Dijon, France; 12 Service de Réanimation, CHR Metz-Thionville, Hôpital de Mercy, France; 13 Médecine Intensive Réanimation, CHU Amiens-Picardie, Amiens, France; 14 Médecine Intensive Réanimation, Hôpital Lapeyronie, CHU Montpellier, Montpellier, France; 15 Département d’Anesthésie-Réanimation, Hôpital de la Conception, AP-HM, Marseille, France; 16 Service de Médecine Intensive Réanimation R3S, La Pitié-Salpêtrière, AP-HP, Paris, France; 17 Réanimation des Urgences et déchocage, Hôpital de la Timone, AP-HM, Marseille, France; 18 Réanimation polyvalente, CH Argenteuil, Argenteuil, France; 19 Médecine Intensive Réanimation, CHU Caen-Normandie, Caen, France; 20 Médecine Intensive Réanimation, CHRU de Tours, Tours, France; 21 Service de Médecine Intensive Réanimation, Hôpital Bichat, AP-HP, Paris, France; 22 Service de Réanimation Médicale, Hôpital Saint Antoine, AP-HP, Paris, France; 23 Service de Réanimation Médicale, Hôpital Européen Georges Pompidou, AP-HP, Paris, France; 24 Médecine Intensive Réanimation, CHU de Rennes, Rennes, France; 25 Service de Réanimation Polyvalente, Hôpital Nord, AP-HM, Marseille, France; 26 Médecine Intensive Réanimation, Hôpital l’Archet 2, CHU de Nice, Nice, France; 27 Anesthésie-Réanimation, Hôpital Privé de l’Ouest Parisien, Trappes, France; 28 Réanimation Polyvalente et surveillance Continue, CH Aix, Aix en Provence, France; 29 Service de Réanimation Médico-chirurgicale, Hôpital Avicenne, AP-HP, Bobigny, France; 30 Service de Médecine Intensive Réanimation, Hôpital Charles Nicolle, CHU de Rouen, Rouen, France; 31 Médecine Intensive-Réanimation, CHU de Lille, INSERM U1285, Université de Lille, CNRS, UMR 8576 – UGSF, Lille, France; 32 Service de Réanimation Polyvalente, CH Toulon Saint Musse, Toulon, France; 33 Service de Médecine Intensive Réanimation, Hôpital Louis Mourier, AP-HP, Colombes, France; 34 Réanimation et Surveillance Continue, CHU de Toulouse Purpan, Toulouse, France; 35 Service de Réanimation, Institut Paoli-Calmette, Marseille, France; 36 Service d’Anesthésie Réanimation et Surveillance Continue, Hôpital de la Timone, AP-HM, Marseille, France; 37 Réanimation Médicale, CHU Clermont-Ferrand, Clermont-Ferrand, France; 38 Médecine Intensive Réanimation Hôpital Saint-Louis, AP-HP, Paris, France; 39 Réanimation, Hôpital Foch, Suresnes, France; 40 Réanimation Polyvalente, CH Portes de Provence, Montélimar, France; 41 Médecine Intensive Réanimation, Nouvel Hôpital Civil, CHU Strasbourg, Strasbourg, France; 42 GRC SoLID; Assistance Publique – Hôpitaux de Paris, Hôpital Tenon, DMU APPROCHES, Service de Médecine Intensive Réanimation, CRSA UMRS_938 INSERM Team 5PMed, Sorbonne Université, Paris, France

**Keywords:** Acute kidney injury, intensive care, legionnaires’ disease, legionella, rhabdomyolysis, tubular Injury

## Abstract

**Introduction:**

Legionnaires’ disease (LD) can cause severe pneumonia, requiring intensive care and frequently associated with multiorgan failure. The mechanisms and prognosis of kidney complications in LD remain poorly characterized. We aimed to describe the incidence, clinical characteristics, and prognosis of acute kidney injury (AKI) in patients who are critically ill with LD and identify factors associated with AKI in a multicenter cohort.

**Methods:**

This multicenter retrospective cohort study was conducted in 39 intensive care units (ICUs) in France between 2012 and 2024. Inclusion criteria were a diagnosis of LD requiring invasive mechanical ventilation.

**Results:**

A total of 561 patients were included. Patients exhibited high severity, with a mean sequential organ failure assessment score on D1 of 7.9; 69% had severe acute respiratory distress syndrome (ARDS) and 81% with septic shock. The incidence of AKI was 74% in ICU, with 44% requiring renal replacement therapy (RRT). Rhabdomyolysis (16%), hematuria (52%), and proteinuria (mean 1.49 g/l) were commonly observed. Rhabdomyolysis was an independent risk factor for AKI and RRT requiremen—adjusted odds ratio [aOR], 3.99 [1.59–10.00]; *P =* 0.003 and aOR, 4.07 [2.17–7.63]; *P <* 0.001, respectively. RRT requirement was associated with significantly increased mortality—aOR, 2.49 [1.27–4.87]; *P =* 0.008 at D28, pLog-rank < 0.001. In an exploratory analysis, dual anti-Legionella antibiotic therapy, administered in 87% of cases, was independently associated with lower day-28 mortality (aOR, 0.27; [95% CI, 0.13–0.58]; *P =* 0.001).

**Conclusions:**

AKI appears to be highly prevalent in patients with severe LD, with a high frequency of rhabdomyolysis and proteinuria, suggesting marked kidney involvement. Rhabdomyolysis was independently associated with AKI onset and RRT requirement. AKI was associated with significantly increased mortality, whereas dual anti-Legionella therapy was protective.

## Graphical abstract

**Figure:**
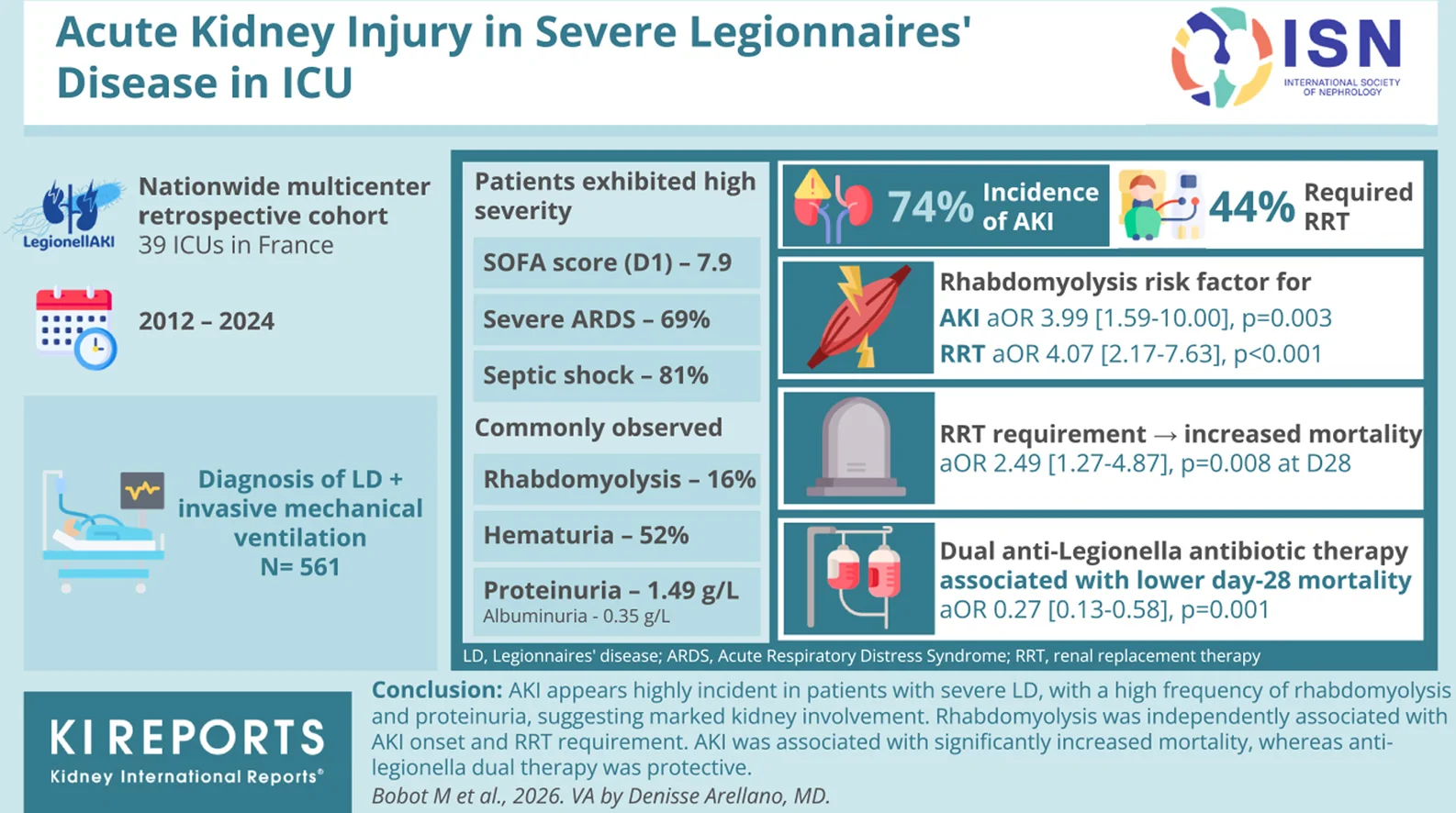

LD, first identified in 1970, is caused by the *Legionella* species^1^ and can be responsible for severe pneumonia progressing to ARDS. Although it is a rare disease, its incidence is increasing, ranging in the post-COVID era from 2 to 3 cases per 100,000 inhabitants in Western countries.^2^ Among hospitalized patients, up to a quarter require ICU admission, mainly for respiratory failure, and 6% to 8% die.^3^^,^^4^

LD is also associated with several extrapulmonary complications.^5^ Kidney involvement in particular has classically been described, especially in the most severely ill patients. Depending on the population and diagnostic criteria, AKI has been diagnosed in up to 78% of patients in cohorts of critically ill adults with LD. Approximately half of these patients required renal replacement therapy during their ICU stay.^6^, ^7^, ^8^ However, the precise pathophysiologic mechanisms of AKI remain unclear.

The *Legionella Pneumophila* (serotype 1) antigen can usually be isolated in the urine; however, there are very few histopathologic descriptions of kidney involvement. The few published cases of kidney biopsies in patients with LD found heterogeneous pathologic findings including tubulointerstitial nephritis, acute tubular necrosis, and glomerulonephritis.^9^, ^10^, ^11^ Legionella-associated myositis is one of the oldest etiological hypotheses,^12^^,^^13^ on the basis of several reported cases of rhabdomyolysis inducing tubular toxicity and AKI.^14^^,^^15^ Other factors, such as hypovolemia, caused by digestive disorders, septic shock, hypoperfusion-driven acute tubular necrosis, or drug nephrotoxicity may be involved.^16^ Underlying medical conditions, such as chronic kidney disease (CKD) or congestive heart failure, may also play a role. Finally, direct pathogenicity of the bacteria cannot be excluded, as Shah *et al.*^17^ detected *Legionella pneumophila* in a kidney biopsy using immunofluorescence staining. The risk factors and clinical outcomes of LD-associated AKI remain poorly understood, irrespective of the underlying mechanisms. LD-associated AKI may be associated with higher mortality,^6^ but large cohorts are lacking to draw definitive conclusions.

To gain insight into this common complication of a rare infectious disease, we designed a multicenter retrospective study focusing on the most severely ill population. In a large cohort of ICU adults with LD requiring invasive mechanical ventilation, we aimed to describe the incidence of AKI and identify its risk factors. We also sought to explore its pathophysiologic mechanisms and assess its prognostic impact and clinical outcomes.

## Methods

### Study Design and Patient Selection

We performed a 12-year retrospective multicenter study in 39 ICUs in continental France. From January 1, 2012, to January 1, 2024, we screened all adult patients (age ≥ 18 years) admitted to the participating ICUs for community-acquired pneumonia because of LD requiring invasive mechanical ventilation (within 72 hours of ICU admission). Exclusion criteria were pre-existing kidney failure (defined as estimated glomerular filtration rate < 15 mL/min per 1.73 m^2^ or the need for chronic dialysis), death within 24 hours of ICU admission, admission from another ICU with missing baseline data, and missing data regarding kidney function in the ICU.

### Data Collection

Investigators extracted predefined clinical, biological, microbiological, and radiological data from the medical charts for each recruited patient and recorded the data on a case report form. Enrolled patients were followed until hospital discharge. Two investigators centrally reviewed all anonymized case report forms to minimize missing data. A nephrologist centrally reviewed histological data from kidney biopsies or autopsies, when performed.

### Definitions

LD was defined by at least 1 positive test in the ICU (or within 72 hours before ICU admission) among urine antigen testing, polymerase chain reaction testing of any lower respiratory tract specimen, documentation of any *Legionella* species by bacterial culture of a lower respiratory tract specimen, and a serum antibody detection test showing the appearance of IgM on paired sera.

AKI and its severity were defined according to the 2012 Kidney Disease: Improving Global Outcome (KDIGO) criteria as an increase in serum creatinine by ≥ 26.5 μmol/l within 48 hours or to ≥ 1.5 times the baseline value, or urine volume < 0.5 mL/kg/h for 6 hours^18^ during the ICU stay. Baseline kidney function was defined using the most recent serum creatinine value available before hospital admission. When no preadmission creatinine was available and the admission serum creatinine and clinical features were not suggestive of AKI, the admission creatinine was used as the baseline value. No back-calculation or imputation methods were applied for patients without an available preadmission creatinine.

Severity was evaluated on day 1 (D1) of the ICU admission. Urinary parameters were collected on spot urine at D1. Rhabdomyolysis was defined as a serum creatine kinase (CK) level > 5000 IU/L.^19^

Additional definitions are provided in Supplementary Methods.

### End Points

The primary end point was new-onset AKI, defined as the occurrence of AKI according to KDIGO criteria at any time during the ICU stay. Secondary end points included AKI severity, the need for RRT and D28 mortality.

### Statistical Analysis

Quantitative variables were expressed as means and standard deviations, and their distribution was compared using the Student’s *t*-test or the nonparametric Mann-Whitney U test. Categorical variables were expressed as counts (%) and their distribution was compared using the chi-square test or the nonparametric Fisher exact test. All tests were 2-tailed. Missing data were not imputed. Analyses were performed using available-case (complete-case) data for each variable. The number of missing values for each variable was reported in the tables.

Comparisons were conducted on the basis of the primary end point. Subjects with new-onset AKI during the ICU stay and those without were compared for demographics, disease history, paraclinical findings, ICU management, and hospital course. Sensitivity analyses were performed by stratifying the population according to RRT requirement and D28 mortality.

Multivariable logistic regression models were used to identify factors associated with AKI, RRT requirement, and D28 mortality. Variables were selected using a backward stepwise procedure on the basis of variables associated with the outcome in univariate analysis (*P <* 0.10) and clinically relevant variables. Collinearity was assessed using variance inflation factors. Long-term survival was estimated by Kaplan-Meier analysis and compared using the log-rank test. Analyses were performed using SAS JMP Pro 14.0 (SAS Institute Inc., Cary, NC) and GraphPad Prism 8.0.1 (GraphPad Prism, La Jolla, CA). A *P* value < 0.05 was considered statistically significant.

## Results

### Population and Baseline Characteristics

Between January 2012 and January 2024, 561 patients were included (male, 73%; age 61 ± 13.2 years; body mass index, 27.2 ± 5.8 kg/m^2^; current smoker, 51%; immunocompromised status, 23%). Table 1 summarizes the baseline characteristics. The most frequent comorbidities were hypertension (42%), dyslipidemia (27%), alcohol abuse (27%), and diabetes (21%). A total of 64 patients (11%) had CKD. Almost one-third of the population (31%) was chronically treated with inhibitors of the renin-angiotensin system, either angiotensin-converting enzyme inhibitors or angiotensin II receptor blockers. LD was microbiologically confirmed by a positive urinary antigen test, bacterial culture, PCR, and serology in 83%, 21%, 17%, and 2% of patients, respectively. In several cases, the diagnosis was established using multiple diagnostic methods (*n* = 122 [22%] with at least 2 positive methods). On D1, 81% of patients required mechanical ventilation, with a mean PaO_2_/FiO_2_ ratio of 114 ± 56.5 mmHg, and 62% had septic shock. The mean simplified acute physiology score II was 50 ± 17.

**Table 1:** Baseline characteristics of 561 patients admitted to the ICU for LD requiring mechanical ventilation, according to new-onset AKI

Characteristics	Missing data	Overall population (*n* = 561)	AKI (*n* = 416)	No AKI (*n* = 145)
Sex, male	0	412 (73.4)	307 (73.8)	105 (72.4)
Age (y)	0	61 ± 13.2	62.3 ± 12.9	57.1 ± 13.1
Obesity	0	132 (23.5)	103 (24.8)	29 (20.0)
Hypertension	0	233 (41.5)	187 (44.9)	46 (31.7)
Diabetes	0	120 (21.4)	88 (21.2)	32 (22.1)
Current smoking	0	284 (50.6)	192 (46.2)	92 (63.5)
Alcohol abuse	0	152 (27.0)	102 (24.5)	50 (34.5)
Coronary artery disease	0	86 (15.3)	70 (16.8)	16 (11)
Atrial fibrillation	0	75 (13.3)	69 (16.6)	6 (4.1)
Congestive heart failure	0	62 (11.0)	58 (13.9)	4 (2.8)
Peripheral artery disease	0	58 (10.3)	51 (12.3)	7 (4.8)
COPD	0	74 (13.2)	54 (12.9)	20 (13.8)
Liver cirrhosis	0	18 (3.2)	14 (3.4)	4 (2.8)
Chronic kidney disease	0	64 (11.4)	61 (14.7)	3 (2.1)
Immunocompromised status	0	126 (22.5)	88 (21.2)	38 (26.2)
HIV		11 (2.0)	8 (1.9)	3 (2.1)
Long-term corticosteroids		58 (10.3)	38 (9.1)	20 (13.8)
Solid organ transplantation		15 (2.7)	13 (3.1)	2 (1.4)
Immunosuppressive drugs		82 (14.6)	55 (13.2)	27 (18.6)
Splenectomy		4 (0.7)	2 (0.5)	2 (1.4)
Current active neoplasia		51 (9.1)	37 (8.9)	14 (9.7)
Recent chemotherapy		28 (5.0)	18 (4.3)	10 (6.9)
ACEi or ARB	0	176 (31.4)	146 (35.1)	30 (20.7)
NSAIDs	0	25 (4.4)	21 (5.1)	4 (2.8)
SOFA	43	7.9 ± 3.5	8.47 ± 3.52	6.19 ± 2.77
SAPS II	22	49.7 ± 16.7	52.9 ± 16.5	40.8 ± 14.1
Invasive mechanical ventilation	3	452 (81.0)	340 (82.3)	112 (77.2)
PaO2/FiO2, mmHg	31	114 ± 56	112 ± 55	120 ± 59
Shock	3	344 (61.6)	272 (65.9)	72 (49.7)
Glasgow coma scale	9	14.0 ± 2.5	13.9 ± 2.7	14.3 ± 1.8
Urea, mmol/l	13	13.1 ± 8.5	15.2 ± 8.7	6.9 ± 3.4
Serum creatinine, μmol/l	7	161.8 ± 138.6	193.6 ± 147.6	70.7 ± 25.6
CK, IU/L	185	4007 ± 18323	4974 ± 20969	1020 ± 2015
Lactate, mmol/l	40	2.40 ± 1.94	2.60 ± 2.12	2.10 ± 1.14
Leukocytes, G/L	17	15.4 ± 23.2	15.9 ± 24.6	13.9 ± 18.9
CRP, mg/l	170	396 ± 149	407 ± 149	363 ± 144
Proteinuria, g/l	435	1.49 ± 1.55	1.38 ± 1.38	1.97 ± 1.98
Albuminuria, mg/l	539	348 ± 511	374 ± 531	98 ± 16
Hematuria	306	133 (52.1)	105 (52.5)	28 (50.9)
Glycosuria	462	44 (44.4)	37 (46.8)	7 (35.0)

ACEi, angiotensin converting enzyme inhibitors; AKI, acute kidney injury; ARB, angiotensin II receptor blockers; COPD, chronic obstructive pulmonary disease; CK, creatine kinase; CRP, C-reactive protein; HIV, human immunodeficiency virus; ICU, intensive care unit; NSAIDs, nonsteroidal anti-inflammatory drugs; PaO_2_/FiO_2_, ratio of partial pressure of arterial oxygen (PaO_2_) to the fraction of inspired oxygen (FIO_2_); SAPS II, simplified acute physiology score II; SOFA, sequen organ failure assessment.

Patients were compared according to the new onset AKI during the ICU stay, according to the 2012 KDIGO.

Data are *n* (%) or mean ± SD.

### Management, ICU Course, and Outcomes

Macrolides and fluoroquinolones were the most prescribed antibiotics, used in 92% of the patients, whereas 87% received dual antibiotic therapy, mainly consisting of macrolides and fluoroquinolones (Table 2). During their ICU stay, most patients (69%) had severe ARDS, 20% received inhaled nitric oxide, and 13% required extracorporeal membrane oxygenation. Of the 454 patients (80%) who experienced shock during their ICU stay, the mean maximum noradrenaline dose was 5.22 ± 7.27 mg/h. The median duration of mechanical ventilation was 11 days (6;18) (Table 3). Median ICU and hospital lengths of stay were 15 days (10;24) and 26 days (17;40), respectively. Median follow-up after hospital admission was 205 days (25;1370). The in-ICU mortality rate was 23%.

**Table 2:** In-ICU management, course, and outcomes of 561 patients admitted to the ICU for LD requiring mechanical ventilation, according to new-onset AKI

Management and outcomes	Missing data	Overall population (*n* = 561)	AKI (*n* = 416)	No AKI (*n* = 145)	*P-*value
Anti-Legionella antibiotics					
Macrolide	4	510 (91.6)	378 (91.7)	132 (91.0)	0.791
Fluoroquinolone	4	514 (92.2)	381 (92.5)	133 (91.7)	0.771
Rifampicin	4	66 (11.8)	49 (11.9)	17 (11.7)	0.957
Dual antibiotic therapy	4	487 (87.4)	359 (87.1)	128 (88.3)	0.722
Organ failures and supports during the ICU stay					
ARDS Severity	15				0.004
Mild		9 (1.6)	5 (1.3)	4 (2.8)	
Moderate		159 (29.1)	102 (25.2)	57 (40.1)	
Severe		375 (68.7)	295 (73.0)	80 (56.3)	
Worst PaO2/FiO2 ratio, mmHg	40	93 ± 41	90 ± 40	101 ± 42	0.009
Highest PEEP, cmH_2_O	86	12.8 ± 3.3	12.9 ± 3.3	12.7 ± 3.2	0.692
Inhaled nitric oxide	0	110 (19.6)	91 (21.9)	19 (13.1)	0.022
ECMO	0	71 (12.7)	63 (15.1)	8 (5.5)	0.003
Shock	0	454 (80.1)	358 (86.1)	96 (66.2)	< 0.001
Maximum norepinephrine, mg/h	164	5.2 ± 7.3	6.2 ± 7.9	1.8 ± 1.6	< 0.001
Corticosteroids	0	111 (19.8)	84 (20.2)	27 (18.6)	0.683
ICU course and outcomes					
Duration of mechanical ventilation	8	11 (6;18)	11 (6;19)	9 (6;13)	< 0.001
Ventilator-free days at D28	1	11.4 ± 9.4	10.0 ± 9.3	15.3 ± 8.5	< 0.001
Duration of ICU stay, d	3	15 (10;24)	17 (10;27)	13 (9;20)	0.002
ICU-free d at D28	0	7.9 ± 7.9	6.7 ± 7.6	11.4 ± 7.6	< 0.001
Hospital length of stay, d	58	26 (17;40)	28 (18;43)	22 (16;32)	0.002
D28 mortality rate	15	116 (21.2)	101 (24.8)	15 (10.9)	0.001
In-ICU mortality rate	1	129 (23.0)	111 (26.7)	18 (12.5)	0.001

AKI, acute kidney injury; ECMO, extra-corporeal membrane oxygenation; ICU: intensive care unit; PaO_2_/FiO_2_, ratio of partial pressure of arterial oxygen (PaO_2_) to the fraction of inspired oxygen (FIO_2_); PEEP, positive end-expiratory pressure.

Patients were compared according to the new onset AKI during the ICU stay, according to the 2012 KDIGO.

Data are *n* (%) or mean ± SD, except for time-dependent variables, which are expressed as median [interquartile range].

**Table 3:** Kidney impairment in 561 patients admitted to the ICU for LD requiring mechanical ventilation, according to new-onset AKI

Management and outcomes	Missing data	Overall population (*n* = 561)	AKI (*n* = 416)	No AKI (*n*= 145)	*P*
Severity of AKI in ICU	0				-
KDIGO 1		110 (19.7)	110 (26.6)	0 (0)	
KDIGO 2		58 (10.4)	58 (14.0)	0 (0)	
KDIGO 3		246 (44.0)	246 (59.4)	0 (0)	
Need for RRT	0	203 (36.1)	203 (48.8)	0 (0)	-
RRT duration (if weaned as ICU discharge)	108	11 [4;22]	11 [4;22]	-	-
Peak serum creatinine, μmol/l	19	245.8 ± 201.1	302.9 ± 204.7	84.7 ± 35.9	< 0.001
Peak urea, mmol/l	21	23.7 ± 14.2	27.9 ± 13.9	11.7 ± 5.7	< 0.001
Peak CK, IU/L	158	5521 ± 24833	6829 ± 28417	1557 ± 4419	0.002
CK > 5000 IU/L	156	62 (15.9)	56 (18.4)	6 (6.0)	0.003
Peak LDH, U/L	238	2067 ± 9628	2510 ± 11086	743 ± 730	0.014
Exposure to aminoglycosides within ICU	0	125 (22.3)	102 (24.5)	23 (15.9)	0.031
Duration, d	19	1 [1;1]	1 [1;1]	1 [1;1]	0.784
Exposure to vancomycin within ICU	0	55 (9.8)	43 (10.3)	12 (8.3)	0.472
Duration, d	10	4 [2;7]	3 [2;7]	5 [2;13]	0.318
Exposure to iodinated contrast media within ICU	21	245 (45.7)	180 (45)	67 (47.9)	0.560
Weaned from RRT at ICU discharge	108	99 (17.6)	99 (23.8)	-	-
Serum creatinine at ICU discharge, μmol/l	136	101.6 ± 96.3	122.6 ± 109	55.1 ± 18	< 0.001
Serum creatinine at hospital discharge, μmol/l	187	93.9 ± 72.4	106.4 ± 79	62.5 ± 38	< 0.001

AKI, acute kidney injury; CK, creatine kinase; ICU, intensive care unit; KDIGO, Kidney Disease Improving Global Outcome; LDH; lactate dehydrogenase; RRT, renal replacement therapy.

Patients were compared according to the new onset AKI during the ICU stay, according to the 2012 KDIGO.

Data are *n* (%) or mean ± standard deviation, except for time-dependent variables, which are expressed as median [interquartile range].

### Kidney Impairment

**Figure 1:**
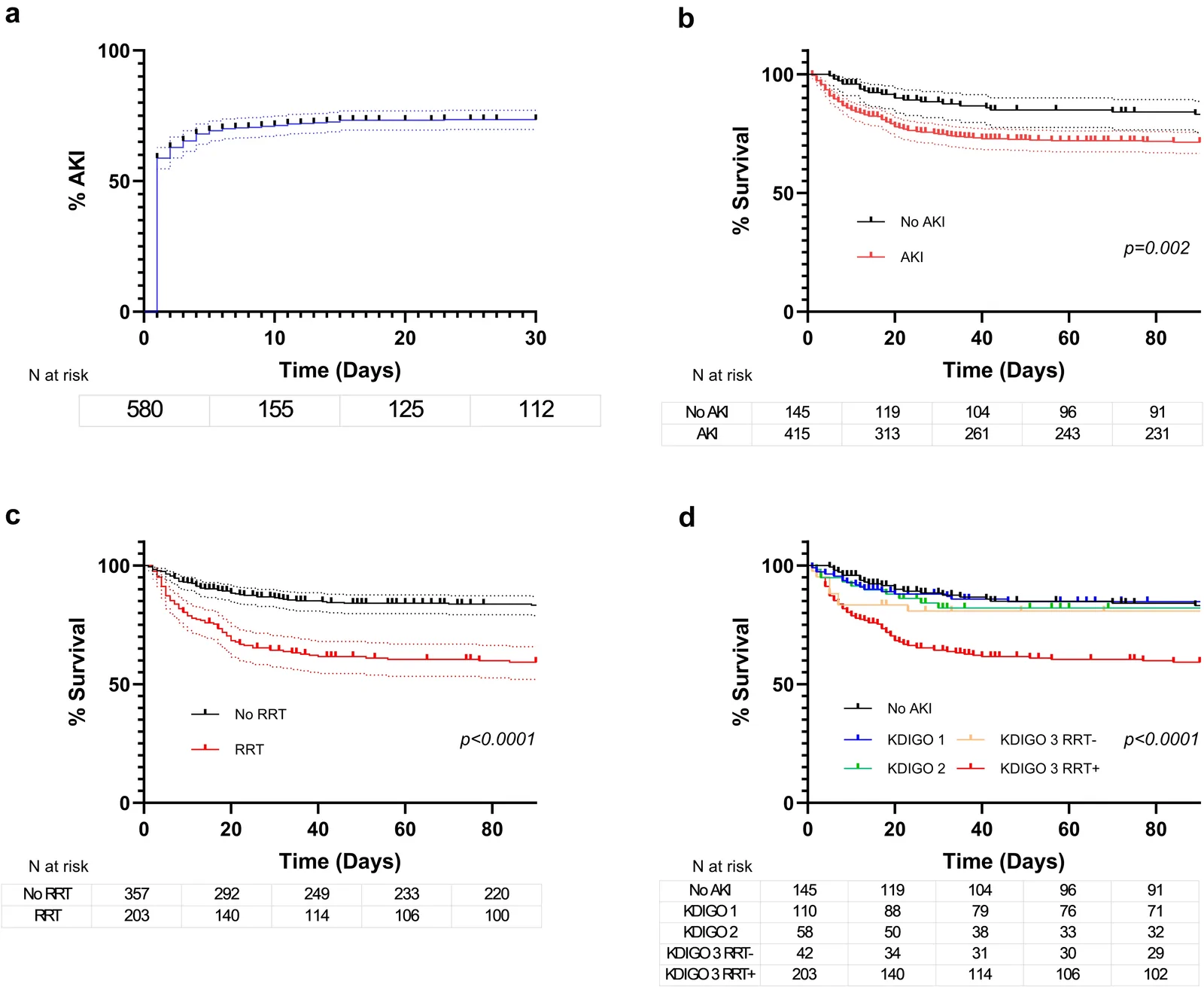
Survival analyses. (a) AKI onset from ICU admission. (b) Patient survival according to AKI onset. (c) Patient survival according to RRT requirement. (d) Patient survival according to AKI severity. AKI, acute kidney Injury; KDIGO, Kidney Disease Improval Global Outcomes; RRT, renal replacement therapy.

On ICU admission, 63% of the patients had AKI (Figure 1a), with a mean creatinine level of 161.8 ± 138.6 μmol/l. Mean proteinuria was 1.49 ± 1.55 g/l, with a proteinuria-to-creatininuria ratio of 1.40 ± 1.24 g/g, and mean albuminuria was 348.4 ± 511.2 mg/l, with an albuminuria-to-creatininuria ratio of 231.8 ± 267.1 mg/g. Hematuria and glycosuria were documented in 133 and 44 patients, respectively. During the ICU stay, 74% of patients experienced AKI, more than half of whom had severe AKI (KDIGO 3). Focusing on risk factors for kidney injury, 16% of patients had blood CK levels greater than 5000 IU/L during their ICU stay. In addition, 22% of the patients were treated with aminoglycosides (median 1 day [1;1]), 10% received vancomycin (4 days [2;7]), and 46% received iodinated contrast media. RRT was required in 49% (203/416) of patients with new-onset AKI, with a median RRT duration of 11 days [4;22]. Only half of them (99/203) were weaned from RRT at ICU discharge. Mean creatinine levels at ICU and hospital discharge were 101.6 ± 96.3 μmol/l and 93.9 ± 72.4 μmol/l, respectively.

Only 9 patients had available renal pathology. Of these, 7 underwent kidney biopsy and 2 underwent autopsy. Lesions of acute tubular necrosis were observed in all cases. In addition, various abnormalities were documented, including intratubular precipitation of calcium oxalate crystals in 2 cases, acute tubulointerstitial nephritis with granulomatous lesions in 1 case, and vancomycin-cast nephropathy and mesangial and Bowman’s capsule enlargement in a patient with established diabetic nephropathy.

### Risk Factors of New-Onset AKI

In univariate analysis, several demographic and medical history variables were associated with new-onset AKI, including age, hypertension, arrhythmia, chronic heart failure, peripheral vascular disease, and pre-existing CKD. Patients with new-onset AKI had higher (sequential organ failure assessment and simplified acute physiology score II) on D1 than their counterparts and experienced a more prolonged and fatal course in the ICU. They required a longer duration of mechanical ventilation and had longer ICU and hospital lengths of stay, as well as higher mortality rates. During their ICU stay, these patients were more likely to receive extracorporeal membrane oxygenation (15.1% vs 5.5%; *P =* 0.003), inhaled nitric oxide (21.9% vs 13.1%; *P =* 0.022), and aminoglycosides (24.5% vs 18.6%; *P =* 0.031). They also experienced rhabdomyolysis more frequently (CK > 5000 IU/L, 18.4% vs 6%; *P =* 0.003), had higher mean CK levels (6829 ± 28417 vs 1557 ± 4419 IU/L; *P =* 0.002) and shock (86.1% vs 66.2%; *P <* 0.001), with a higher maximum dose of norepinephrine (6.19 ± 7.9 vs 1.83 ± 1.6 mg/h, *P <* 0.001).

In multivariate analysis, the independent risk factors for new-onset AKI were a history of heart failure (aOR 4.15 [1.18;14.58], *P =* 0.027) and CKD (aOR 6.95 [1.54;31.28], *P =* 0.012), a higher initial simplified acute physiology score II (aOR 1.06 [1.04;1.08], *P <* 0.001), and the presence of rhabdomyolysis (aOR 3.99 [1.59;10.00], *P =* 0.003) (Table 4). Models were additionally adjusted for center; this adjustment did not materially change the results.

**Table 4:** Multivariate analysis of variables associated with new-onset AKI and need for RRT

Risk factors	AKI		Need for RRT	
	aOR(95% CI)	*P*	aOR (95% CI)	*P*
Age (y)	1.00 (0.98; 1.02)^a^	0.985	0.98 (0.96; 0.999)^a^	0.034
SAPS-II at D1	1.06 (1.04; 1.08)^a^	< 0.001	1.06 (1.04; 1.08)^a^	< 0.001
Hypertension	0.93 (0.53; 1.63)	0.788	0.89 (0.53; 1.50)	0.657
Congestive heart failure	4.14 (1.18; 14.58)	0.027	1.17 (0.53; 2.58)	0.694
CKD	6.95 (1.54; 31.28)	0.012	7.74 (3.41; 17.60)	< 0.001
Rhabdomyolysis (CK > 5000 IU/L)	3.99 (1.59; 10.00)	0.003	4.07 (2.17; 7.63)	< 0.001

aOR, adjusted odds ratio; AKI, acute kidney injury; CK, creatine kinase; CKD, chronic kidney disease; ECMO, extra-corporeal membrane oxygenation; ICU, intensive care unit; RRT: renal replacement therapy; SAPS-II, simplified acute physiology score II.

a For a one-unit increase.

In a sensitivity analysis, patients were stratified according to RRT requirement during the ICU stay (Supplementary Tables S1–S3). In univariate analysis, patients requiring RRT had more comorbidities, were more severely ill at baseline, and experienced rhabdomyolysis more frequently (26.1% vs 8.1%, *P <* 0.001). These patients also had a more prolonged and fatal course within the ICU. In multivariate analysis, factors independently associated with the need for RRT were similar to those associated with new-onset AKI (Table 4). In addition, adjustment for year of ICU admission did not substantially modify the associations with AKI and RRT requirement (Supplementary Table S4). These analyses confirmed the robustness of the associations observed in the primary analysis.

### Exploratory Analyses on D28 Mortality

Finally, patients were stratified according to D28 mortality (Supplementary Tables S5–S7). In univariate analysis, deceased patients had a lower frequency of dual antibiotic therapy (78.6% vs 89.5%, *P =* 0.002) and quinolone use (84.8% vs 94.2%; *P =* 0.001) with to survivors. In multivariate analysis, a history of active neoplasia (aOR, 4.41 [1.92–10.09]; *P =* 0.001) and cirrhosis (aOR, 4.34 [1.03–18.30]; *P* *=* 0.045), maximum dose of noradrenaline (aOR 1.12 [1.06;1.18]; *P <* 0.001), and the need for RRT (aOR 2.49 [1.27–4.87]; *P =* 0.008) were independently associated with D28 mortality. Conversely, obesity (aOR, 0.45 [0.21–0.98]; *P =* 0.045) and dual antibiotic therapy (aOR, 0.27 [0.13;0.58]; *P =* 0.001) were associated with lower D28 mortality (Table 5). Results were similar after adjustment for center, and additional adjustment for year of ICU admission did not modify these results (Supplementary Table S8).

**Table 5:** Risk factors of mortality at D28, multivariate analysis

Risk factors	Mortality	
	aOR (95% CI)	*P*
Age (y)	1.02 (0.99; 1.05)^a^	0.109
SAPS-II at D1	1.02 (0.99; 1.04)^a^	0.130
Maximum dose of norepinephrin (mg/h)	1.12 (1.06; 1.18)^a^	< 0.001
Worst PaO2/FiO2	0.99 (0.98; 1.00)^a^	0.113
CKD	0.93 (0.38; 2.29)	0.880
Obesity	0.45 (0.21; 0.98)	0.045
Chronic respiratory disease (non-COPD)	2.13 (0.82; 5.49)	0.118
Current neoplasia	4.41 (1.92; 10.09)	0.001
RRT	2.49 (1.27; 4.87)	0.008
Atrial fibrillation	1.54 (0.65; 3.67)	0.330
Liver cirrhosis	4.34 (1.03; 18.30)	0.045
Dual antibiotic therapy	0.27 (0.13; 0.58)	0.001

aOR, adjusted odds ratio; COPD, chronic obstructive pulmonary disease; CKD, chronic kidney disease; RRT, renal replacement therapy; SAPS-II, simplified acute physiology score II.

a For a one-unit increase.

Survival analyses confirmed these findings, with higher long-term mortality in patients with new-onset AKI (Figure 1b) and those requiring RRT (Figure 1c). Mortality increased with AKI severity (Figure 1d), particularly in patients with AKI KDIGO 3 who required RRT.

## Discussion

We investigated kidney involvement in the largest multicenter cohort to date of patients admitted to the ICU for severe pneumonia caused due to *Legionella*. Our findings provide clinically relevant insights into the presentation, complications, and management of severe LD.

Our work highlights a very high incidence of AKI associated with severe LD (74%), which is much higher than the figures found in large cohort studies of all-cause community-acquired pneumonia, ranging from 37% to 57%.^20^^,^^21^ Our findings suggest particular features of kidney involvement in LD. First, a high proportion of patients exhibited significant proteinuria (mean proteinuria, 1.49 ± 1.55 g/l). Among the small subset of patients with concomitant albuminuria measurements, the low urinary albumin-to-protein ratio indicated a predominantly tubular pattern. Hematuria was also frequent in our cohort, though its interpretation in the ICU can be challenging, given the common use of bladder catheters possibly increasing its frequence. Finally, there was a strong association between rhabdomyolysis and both AKI and the need for RRT. Collectively, these data may further substantiate the hypothesis of specific kidney involvement in LD.

We found that 16% of patients in our cohort exhibited significant rhabdomyolysis (CK > 5000 IU/L). Previous reports have described rhabdomyolysis as a frequent complication of severe LD; however, its true incidence remains uncertain because available data are mainly limited to small case series and because of the variability in CK threshold values chosen to define rhabdomyolysis across studies. A 1983 prospective study found that 25 of 32 patients (78%) with LD had elevated CK levels (> 175 IU/L),^22^ which is consistent with our findings (mean peak CK level during the ICU stay was 677 IU/L). These results support the hypothesis of a specific muscle toxicity of LD. Early identification of rhabdomyolysis may enable adaptation of fluid therapy to potentially improve kidney prognosis in these patients.

Patient characteristics and associated comorbidities were consistent with those described in recent studies.^23^, ^24^, ^25^ Patients in our cohort exhibited a high level of severity and multiorgan failure.

In this study, previous CKD was associated with AKI and the need for RRT, as were chronic heart failure and the initial overall severity of patients. Iatrogenicity could also play an important role in the onset of AKI. Exposure to inhaled nitric oxide was previously described in patients with COVID-19-related ARDS in the ICU.^26^ However, the observed association between inhaled nitric oxide and AKI may be influenced by confounding by indication and global disease severity. Invasive ventilation may also have contributed in AKI onset.^27^ Although exposure to corticosteroid therapy does not appear to affect kidney impairment during LD, contrary to what has been described in COVID-19-related ARDS,^28^ corticosteroid regimens used in the ICU for septic shock, severe pneumonia, or lung fibroproliferation differ substantially from those used to treat biopsy-proven acute tubulointerstitial nephritis.

Kidney biopsy findings predominantly revealed acute tubular necrosis lesions, likely because of shock severity and drug-induced nephrotoxicity (vancomycin, aminoglycosides), rhabdomyolysis, and possibly direct tubular toxicity of the bacteria. Notably, 2 of 9 patients exhibited intratubular precipitation of calcium oxalate, a finding that has not been previously reported. However, the small number of biopsies performed and the often- delayed timing of biopsies or autopsies preclude definitive conclusions regarding the histological spectrum and pathophysiologic mechanisms of kidney injury in severe LD. Finally, although hyponatremia has been described as a characteristic feature of LD,^29^ significant hyponatremia was not observed in most patients in our cohort.

Although not the primary focus of this study, noteworthy data on mortality in the context of ARDS due to *L. pneumophila* were collected. Our cohort had a 21% mortality rate at D28, which is consistent with previous reports.^3^^,^^8^^,^^23^^,^^30^ The cohort by Cecchini *et al.*^6^ in 2017 reported a 26% mortality rate among 210 critically ill patients with LD in France, but only 69% of patients presented with ARDS. The study also highlighted a potential decrease in mortality over the years,^6^ possibly explained by improved management of ARDS, optimization of ventilation parameters, wider use of prone positioning, and VV- extracorporeal membrane oxygenation (13% of our cohort). However, mortality in LD remains high, reflecting the severity of this pathology in critical care. AKI is commonly associated with mortality in septic shock from all causes.^31^ Severe AKI requiring RRT was strongly associated with mortality in our study (2.5-fold higher).

In recent years, few modifiable factors have been identified to improve mortality associated with LD, as mortality appears to be primarily related to initial severity.^32^ The use of antibiotic therapy in severe legionellosis is also widely debated. Two studies have highlighted the importance of promptly initiating effective antibiotic therapy to reduce patient mortality,^8^^,^^33^ consistent with observations in patients with septic shock.^34^ Finally, Cecchini et al.^6^ showed that the use of fluoroquinolones was independently associated with lower mortality. To our knowledge, our study is the first to identify a significant and independent association between lower mortality and dual anti-Legionella antibiotic therapy. This finding could further support the widespread use of this therapy in France (87% of our cohort) and other Western countries, despite the absence of current recommendations.

There are several limitations to our study that warrant consideration, particularly those inherent to its observational and retrospective design. Patients were included over a period of > 10 years, during which time the quality of intensive care and therapeutic procedures may have evolved, although major changes in the management of severe LD have been limited over this period. There is also a substantial amount of missing data, particularly regarding urinary tests, which were not systematically performed at the beginning of the ICU stay. However, the absence of urinary sampling was uniformly distributed across study centers, highlighting current practices in French ICUs. Finally, although dual anti-Legionella therapy was associated with lower mortality, our study did not capture the specific antibiotic combinations or treatment duration. Whether these factors influence outcomes warrants further investigation.

Our study also has several strengths. To date and to our knowledge, it is the largest multicenter cohort study of critically ill patients with LD, including patients managed in 39 ICUs across France, and the largest cohort describing AKI in patients with severe LD. It provides a robust reflection of practices in French and European ICUs. A large amount of data was collected, allowing for a comprehensive description of the epidemiology of severe LD. Moreover, this is the first multicenter study to specifically describe kidney damage associated with LD.

## Conclusion

Severe LD is associated with a high incidence of AKI, higher than in severe all-cause community-acquired pneumonia, with frequent rhabdomyolysis and proteinuria, suggesting marked kidney involvement. Patients’ initial severity and rhabdomyolysis were independently associated with the onset of AKI and the need for RRT, supporting the central role of rhabdomyolysis in LD-related AKI. Patients with AKI, particularly those requiring RRT, exhibited significantly higher mortality, whereas dual anti-Legionella therapy was protective.

## Appendix

### Members of the LegionellAKI study group:

Laurent Argaud, Romain Arrestier, François Arrive, Damien Barrau, Nacim Benchabane, Mickaël Bobot, Nicolas Bruder, Come Bureau, Julien Carvelli, Emile Charruyer, Khalil Chaibi, Paul Chanareille, Jonathan Chelly, Damien Contou, Cédric Daubin, Nina De Montmollin, Julien Demiselle, Pierre-François Dequin, Julien Dessajan, Paul Dunand, Alexandre Elabbadi, Julien Faraut, Paul Gabarre, Emmanuel Guerot, Pauline Guillot, Camille Haumont, Sami Hraiech, Hervé Hyvernat, Mathieu Jamme, Kada Klouche, Laurent Lefebvre, Marc Leone, Guillaume Louis, Julien Maizel, Julien Moury, Jonathan Nicolas, Saad Nseir, Benoit Painvain, Noémie Peres, Gaël Piton, Jean-Pierre Quenot, Noémie Resseguier, Jean-Damien Ricard, Jean-Christophe Richard, Benjamine Sarton, Luca Servan, Pierre Simeone, Bertrand Souweine, Guillaume Voiriot, Lara Zafrani, and Benjamin Zuber.

## Disclosure

All the authors declared no competing interests.

## Supplementary Material

Supplementary File (PDF) **Supplementary Methods. Supplementary Reference. Table S1.** Baseline characteristics of 561 patients admitted to the ICU for Legionnaire’s disease requiring mechanical ventilation and comparison between patients who needed RRT or not. **Table S2.** In-ICU management, course, and outcomes of 561 patients admitted to the ICU for Legionnaire’s disease requiring mechanical ventilation and comparison between patients who needed RRT or not. **Table S3.** Kidney impairment in 561 patients admitted to the ICU for Legionnaire’s disease requiring mechanical ventilation, according to new-onset AKI and comparison between patients who needed RRT or not. **Table S4.** Sensitivity analysis: Multivariate analysis of variables associated with new-onset AKI and need for renal replacement therapy additionally adjusted for year of ICU admission. **Table S5.** Factors associated with D28 mortality, univariate analysis: baseline characteristics. **Table S6.** Factors associated with D28 mortality, univariate analysis: in-ICU management, course, and outcomes. **Table S7.** Factors associated with D28 mortality, univariate analysis: kidney impairment. **Table S8.** Sensitivity analysis: Multivariate analysis of variables associated with mortality at D28, additionally adjusted for year of ICU admission. STROBE Statement.

## Footnotes

Supplementary File (PDF)
**Supplementary Methods**. **Supplementary Reference**. **Table S1.** Baseline characteristics of 561 patients admitted to the ICU for Legionnaire’s disease requiring mechanical ventilation and comparison between patients who needed RRT or not. **Table S2.** In-ICU management, course, and outcomes of 561 patients admitted to the ICU for Legionnaire’s disease requiring mechanical ventilation and comparison between patients who needed RRT or not. **Table S3.** Kidney impairment in 561 patients admitted to the ICU for Legionnaire’s disease requiring mechanical ventilation, according to new-onset AKI and comparison between patients who needed RRT or not. **Table S4.** Sensitivity analysis: Multivariate analysis of variables associated with new-onset AKI and need for renal replacement therapy additionally adjusted for year of ICU admission. **Table S5.** Factors associated with D28 mortality, univariate analysis: baseline characteristics. **Table S6.** Factors associated with D28 mortality, univariate analysis: in-ICU management, course, and outcomes. **Table S7.** Factors associated with D28 mortality, univariate analysis: kidney impairment. **Table S8.** Sensitivity analysis: Multivariate analysis of variables associated with mortality at D28, additionally adjusted for year of ICU admission. STROBE Statement.
