# Supplementary material for: AKI in Severe Legionnaires’ Disease in Intensive Care Unit
Source: Kidney Int Rep. 2026 Aug 12;11(10):107018. doi: 10.1016/j.ekir.2026.107018 (PMC13579684; doi:10.1016/j.ekir.2026.107018)
Supplement: Supplementary file 1 [file mmc1.pdf]

**Acute kidney injury in severe Legionnaires' Disease in ICU**

**Supplementary Methods**

**Additional Definitions**

Shock was defined as persistent hypotension requiring vasopressors.

ARDS was defined according to the 2012 Berlin criteria (S1).

Severity was evaluated on day 1 (D1) of the ICU stay using the Sepsis-related organ failure assessment (SOFA) score (S2) and Simplified Acute Physiology Score (SAPS II) (S3).

Chronic kidney disease (CKD) was defined according to the 2024 KDIGO definitions.

Immunocompromised status was defined as ongoing immunosuppressive therapy or cytotoxic chemotherapy, solid organ or bone marrow transplantation, HIV-positive status, or intake of > 5 mg daily dose of prednisone equivalent for at least 30 days.

Obesity was defined as a body mass index > 30 kg/m².

Dual antibiotic therapy was defined as concomitant use of two antibiotics effective against LD.

Day 28 (D28) ICU-free days and ventilator-free days represent, respectively, the number of days alive outside the ICU and the number of days alive without invasive mechanical ventilation, measured at D28 after ICU admission.

**Supplementary References:**

S1. ARDS Definition Task Force, Ranieri VM, Rubenfeld GD, Thompson BT, Ferguson ND, Caldwell E, et al. Acute respiratory distress syndrome: the Berlin Definition. JAMA. 2012 June 20;307(23):2526–33.

S2. Vincent JL, Moreno R, Takala J, Willatts S, De Mendonça A, Bruining H, et al. The SOFA (Sepsis-related Organ Failure Assessment) score to describe organ dysfunction/failure. On behalf of the Working Group on Sepsis-Related Problems of the European Society of Intensive Care Medicine. Intensive Care Med. 1996 July;22(7):707–10.

S3. Le Gall JR, Lemeshow S, Saulnier F. A new Simplified Acute Physiology Score (SAPS II) based on a European/North American multicenter study. JAMA. 1993 Dec 22;270(24):2957–63.

**Supplementary Tables**

**Supplementary Table S1. Baseline characteristics of 561 patients admitted to the ICU for Legionnaire's disease requiring mechanical ventilation and comparison between patients who needed RRT or not.**

| | Missing data | Overall population (n = 561) | RRT (n = 203) | No RRT (n = 358) | p |
|---|---|---|---|---|---|
| **Demographics** | | | | | |
| Sex, male | 0 | 412 (73.4) | 153 (75.4) | 259 (72.4) | 0.436 |
| Age (years) | 0 | 61 ± 13.2 | 61.8 ± 12.8 | 60.5 ± 13.4 | 0.270 |
| **Comorbid conditions** | | | | | |
| Obesity | 0 | 132 (23.5) | 50 (24.6) | 82 (22.9) | 0.643 |
| Hypertension | 0 | 233 (41.5) | 88 (43.4) | 145 (40.5) | 0.511 |
| Diabetes | 0 | 120 (21.4) | 45 (22.2) | 75 (20.9) | 0.735 |
| Current smoking | 0 | 284 (50.6) | 82 (40.4) | 202 (56.4) | 0.001 |
| Alcoholism | 0 | 152 (27.0) | 43 (21.2) | 109 (30.5) | 0.018 |
| Coronary artery disease | 0 | 86 (15.3) | 37 (18.2) | 49 (13.7) | 0.152 |
| Atrial fibrillation | 0 | 75 (13.3) | 31 (15.3) | 44 (12.3) | 0.319 |
| Congestive heart failure | 0 | 62 (11.0) | 30 (14.8) | 32 (8.9) | 0.034 |
| Peripheral artery disease | 0 | 58 (10.3) | 54 (12.9) | 38 (10.6) | 0.776 |
| COPD | 0 | 74 (13.2) | 26 (12.8) | 48 (13.4) | 0.840 |
| Liver cirrhosis | 0 | 18 (3.2) | 6 (2.9) | 12 (3.4) | 0.798 |
| Chronic kidney disease | 0 | 64 (11.4) | 41 (20.2) | 23 (6.4) | < 0.001 |
| Immunocompromised status | 0 | 126 (22.5) | 52 (25.6) | 74 (20.7) | 0.177 |
| HIV | | 11 (2.0) | 4 (2.0) | 7 (2.0) | 1.000 |
| Long-term corticosteroids | | 58 (10.3) | 23 (11.3) | 35 (9.8) | 0.561 |
| Solid organ transplantation | | 15 (2.7) | 10 (4.9) | 5 (1.4) | 0.013 |
| Immunosuppressive drugs | | 82 (14.6) | 28 (13.8) | 54 (15.1) | 0.678 |
| Splenectomy | | 4 (0.7) | 2 (1.0) | 2 (0.6) | 0.623 |
| Current active neoplasia | | 51 (9.1) | 19 (9.4) | 32 (8.9) | 0.868 |
| Recent chemotherapy | | 28 (5.0) | 10 (4.9) | 18 (5.0) | 0.958 |
| **Drug exposure during the 14 days before ICU admission** | | | | | |
| ACEi or ARB | 0 | 176 (31.4) | 68 (33.5) | 108 (30.2) | 0.414 |
| NSAIDs | 0 | 25 (4.4) | 8 (8.9) | 17 (4.8) | 0.656 |
| **Severity during the first day in ICU** | | | | | |
| SOFA | 43 | 7.9 ± 3.5 | 9.6 ± 3.5 | 6.9 ± 3.1 | < 0.001 |
| SAPS II | 22 | 49.7 ± 16.7 | 57.2 ± 16.0 | 45.5 ± 15.6 | < 0.001 |
| Invasive mechanical ventilation | 3 | 452 (81.0) | 166 (83.0) | 286 (79.9) | 0.369 |
| PaO2/FiO2, mmHg | 31 | 114 ± 56 | 112 ± 58 | 115 ± 55 | 0.585 |
| Shock | 3 | 344 (61.6) | 144 (71.6) | 200 (56.0) | < 0.001 |
| Glasgow coma scale | 9 | 14.0 ± 2.5 | 13.9 ± 2.9 | 14.1 ± 2.2 | 0.466 |
| **Laboratory findings during the first day in ICU** | | | | | |
| Urea, mmol/L | 13 | 13.1 ± 8.5 | 17.6 ± 9.5 | 10.5 ± 6.7 | < 0.001 |
| Serum creatinine, µmol/L | 7 | 161.8 ± 138.6 | 252.2 ± 183.0 | 111.3 ± 64.4 | < 0.001 |
| CK, IU/L | 185 | 4007 ± 18323 | 7970 ± 29004 | 1547 ± 2889 | 0.009 |
| Lactate, mmol/L | 40 | 2.40 ± 1.94 | 2.68 ± 2.44 | 2.31 ± 1.58 | 0.064 |
| Leukocytes, G/L | 17 | 15.4 ± 23.2 | 18.3 ± 33.9 | 13.8 ± 14.1 | 0.083 |
| CRP, mg/L | 170 | 396 ± 149 | 428 ± 134 | 379 ± 154 | 0.001 |
| Proteinuria, g/L | 435 | 1.49 ± 1.55 | 1.67 ± 1.65 | 1.36 ± 1.42 | 0.271 |
| Albuminuria, mg/L | 539 | 348 ± 511 | 410 ± 590 | 259 ± 385 | 0.476 |
| Hematuria | 306 | 133 (52.1) | 49 (52.7) | 84 (51.9) | 0.898 |
| Glycosuria | 462 | 44 (44.4) | 24 (51.1) | 20 (35.1) | 0.029 |

Patients were compared according to the new onset AKI during the ICU stay, according to the 2012 Kidney Disease Improving Global Outcome (KDIGO).

Abbreviations: ACEi: angiotensin converting enzyme inhibitors, AKI: acute kidney injury, ARB: angiotensin II receptor blockers, COPD: chronic obstructive pulmonary disease, CK: creatine kinase, CRP: C-reactive protein, HIV: human immunodeficiency virus, ICU: intensive care unit, NSAIDs: non-steroidal anti-inflammatory drugs, $PaO_2/FiO_2$: ratio of partial pressure of arterial oxygen ($PaO_2$) to the fraction of inspired oxygen ($FIO_2$), RRT: Renal Replacement Therapy, SAPS II: Simplified Acute Physiology Score II, SOFA: Sequential Organ Failure Assessment.

Data are n (%) or mean ± standard deviation.

**Supplementary Table S2. In-ICU management, course, and outcomes of 561 patients admitted to the ICU for Legionnaire's disease requiring mechanical ventilation and comparison between patients who needed RRT or not.**

| | Missing data | Overall population (n = 561) | RRT (n = 203) | No RRT (n = 358) | p |
|---|---|---|---|---|---|
| **Anti-Legionella antibiotics** | | | | | |
| Macrolide | 4 | 510 (91.6) | 187 (93.0) | 323 (90.7) | 0.347 |
| Fluoroquinolone | 4 | 514 (92.2) | 188 (93.5) | 326 (91.6) | 0.405 |
| Rifampicin | 4 | 66 (11.8) | 29 (14.4) | 37 (10.4) | 0.157 |
| Dual antibiotic therapy | 4 | 487 (87.4) | 182 (90.6) | 305 (85.7) | 0.096 |
| **Organ failures and supports during the ICU stay** | | | | | |
| ARDS Severity | 15 | | | | < 0.001 |
| Mild | | 9 (1.6) | 4 (2.0) | 5 (1.4) | |
| Moderate | | 159 (29.1) | 37 (18.5) | 122 (35.3) | |
| Severe | | 375 (68.7) | 158 (79.0) | 217 (62.7) | |
| Worst PaO2/FiO2 ratio, mmHg | 40 | 93 ± 41 | 85.6 ± 38.8 | 96.9 ± 41.7 | 0.002 |
| Highest PEEP, $cmH_2O$ | 86 | 12.8 ± 3.3 | 13.2 ± 3.4 | 12.6 ± 3.2 | 0.052 |
| Inhaled nitric oxide | 0 | 110 (19.6) | 61 (30.1) | 49 (13.7) | < 0.001 |
| ECMO | 0 | 71 (12.7) | 45 (22.2) | 26 (7.3) | < 0.001 |
| Shock | 0 | 454 (80.1) | 188 (92.6) | 266 (74.3) | < 0.001 |
| Maximum norepinephrine, mg/h | 164 | 5.2 ± 7.3 | 8.0 ± 7.9 | 3.2 ± 6.1 | < 0.001 |
| Corticosteroids | 0 | 111 (19.8) | 84 (20.2) | 27 (18.6) | 0.017 |
| **ICU course and outcomes** | | | | | |
| Duration of mechanical ventilation, days | 8 | 11 [6;18] | 14 [7;25] | 9 [6;15] | < 0.001 |
| Ventilator-free days at D28 | 1 | 11.4 ± 9.4 | 6.3 ± 8.4 | 14.2 ± 8.7 | < 0.001 |
| Duration of ICU stay, days | 3 | 15 [10;24] | 19 [11;32] | 14 [10;21] | < 0.001 |
| ICU-free days at D28 | 0 | 7.9 ± 7.9 | 3.8 ± 6.2 | 10.3 ± 7.7 | < 0.001 |
| Hospital length of stay, days | 58 | 26 [17;40] | 32 [15;51] | 24 [17;34] | < 0.001 |
| D28 mortality rate | 15 | 116 (21.2) | 70 (35.0) | 46 (13.3) | < 0.001 |
| In-ICU mortality rate | 1 | 129 (23.0) | 78 (38.4) | 51 (14.3) | < 0.001 |

Patients were compared according to the new onset AKI during the ICU stay, according to the 2012 Kidney Disease Improving Global Outcome (KDIGO).
AKI: Acute Kidney Injury, ECMO: Extra-Corporeal Membrane Oxygenation, ICU: intensive care unit, $PaO_2/FiO_2$: ratio of partial pressure of arterial oxygen ($PaO_2$) to the fraction of inspired oxygen ($FIO_2$), PEEP: Positive end-expiratory pressure, RRT: Renal Replacement Therapy.
Data are n (%) or mean ± standard deviation, except for time-dependent variables, which are expressed as median [interquartile range].

**Supplementary Table S3. Kidney impairment in 561 patients admitted to the ICU for Legionnaire's disease requiring mechanical ventilation, according to new-onset AKI and comparison between patients who needed RRT or not.**

| | Missing data | Overall population (n = 561) | RRT (n = 203) | No RRT (n = 358) | p |
|---|---|---|---|---|---|
| Severity of AKI in ICU | 0 | | | | - |
| KDIGO 1 | | 110 (19.7) | 0 (0) | 0 (0) | |
| KDIGO 2 | | 58 (10.4) | 0 (0) | 0 (0) | |
| KDIGO 3 | | 246 (44.0) | 203 (100) | 0 (0) | |
| Peak serum creatinine, µmol/L | 19 | 245.8 ± 201.1 | 422.9 ± 211.3 | 145.5 ± 101.4 | < 0.001 |
| Peak urea, mmol/L | 21 | 23.7 ± 14.2 | 33.3 ± 12.5 | 18.2 ± 12.1 | < 0.001 |
| Peak CK, IU/L | 158 | 5521 ± 24833 | 11601 ± 3934 | 1799 ± 3755 | 0.003 |
| CK > 5000 IU/L | 156 | 62 (15.9) | 40 (26.1) | 22 (8.7) | < 0.001 |
| Peak LDH, U/L | 238 | 2067 ± 9628 | 3760 ± 14845 | 942 ± 2308 | 0.034 |
| Exposure to aminoglycosides within ICU | 0 | 125 (22.3) | 55 (27.1) | 70 (19.6) | 0.039 |
| Duration, days | 19 | 1 [1;1] | 1 [1;2] | 1 [1;1] | 0.030 |
| Exposure to vancomycin within ICU | 0 | 55 (9.8) | 27 (13.3) | 28 (7.8) | 0.036 |
| Duration, days | 10 | 4 [2;7] | 3 [2;7] | 4 [2 ;8] | 0.353 |
| Exposure to iodinated contrast media within ICU | 21 | 245 (45.7) | 96 (49.7) | 151 (43.5) | 0.164 |
| Serum creatinine at ICU discharge, µmol/L | 136 | 101.6 ± 96.3 | 186.4 ± 157.3 | 76.9 ± 45.7 | < 0.001 |
| Serum creatinine at hospital discharge, µmol/L | 187 | 93.9 ± 72.4 | 139.8 ± 105.4 | 76.6 ± 44.5 | < 0.001 |

Patients were compared according to the new onset AKI during the ICU stay, according to the 2012 Kidney Disease Improving Global Outcome (KDIGO).
AKI: Acute Kidney Injury, CK: creatinine kinase, ICU: intensive care unit, LDH, lactate dehydrogenase, RRT: Renal Replacement Therapy.
Data are n (%) or mean ± standard deviation, except for time-dependent variables, which are expressed as median [interquartile range].

**Supplementary Table S4: Sensitivity analysis: Multivariate analysis of variables associated with mortality at D28, additionally adjusted for year of ICU admission**

| Risk factors | Mortality | |
|---|---|---|
| | aOR (95%CI) | p |
| Age (years) | 1.02 [0.997 ; 1.05]* | 0.082 |
| SAPS-II at D1 | 1.01 [0.99 ; 1.04]* | 0.150 |
| Maximum dose of norepinephrin (mg/h) | 1.12 [1.06 ; 1.18]* | <0.001 |
| Worst PaO2/FiO2 | 0.99 [0.98 ; 1.00]* | 0.126 |
| Chronic Kidney Disease | 0.93 [0.38 ; 2.29] | 0.877 |
| Obesity | 0.45 [0.21 ; 0.97] | 0.041 |
| Chronic respiratory disease (non-COPD) | 2.09 [0.81 ; 5.38] | 0.136 |
| Current neoplasia | 4.51 [1.97 ; 10.32] | <0.001 |
| RRT | 2.49 [1.27 ; 4.89] | 0.008 |
| Atrial Fibrillation | 1.48 [0.62 ; 3.54] | 0.383 |
| Liver Cirrhosis | 4.56 [1.07 ; 19.41] | 0.040 |
| Dual antibiotic therapy | 0.28 [0.13 ; 0.60] | 0.001 |
| Year of ICU admission | 0.95 [0.86-1.05]* | 0.336 |

* For a one-unit increase.
aOR: adjusted odds ratio, COPD: Chronic obstructive pulmonary disease, ICU: Intensive Care Unit, RRT: Renal Replacement therapy, SAPS-II: Simplified Acute Physiology Score II

**Supplementary Table S5 : Factors associated with D28 mortality, univariate analysis: baseline characteristics**

| | Dead at D28 (n = 116) | Alive at D28 (n = 430) | p |
|---|---|---|---|
| **Demographics** | | | |
| Sex, male | 85 (73.3) | 315 (73.3) | 0.997 |
| Age (years) | 66.4 ± 13.8 | 59.8 ± 12.6 | < 0.001 |
| **Comorbid conditions** | | | |
| Obesity | 19 (16.4) | 110 (25.6) | 0.038 |
| Hypertension | 55 (47.4) | 173 (40.2) | 0.164 |
| Diabetes | 27 (23.3) | 91 (21.2) | 0.624 |
| Current smoking | 38 (32.8) | 236 (54.9) | < 0.001 |
| Alcoholism | 17 (14.7) | 124 (28.8) | 0.002 |
| Coronary artery disease | 24 (20.7) | 60 (13.9) | 0.074 |
| Atrial fibrillation | 29 (25.0) | 46 (10.7) | < 0.001 |
| Congestive heart failure | 17 (14.7) | 44 (10.2) | 0.180 |
| Peripheral artery disease | 13 (11.2) | 44 (10.2) | 0.761 |
| COPD | 17 (14.6) | 55 (12.8) | 0.598 |
| Liver cirrhosis | 8 (6.9) | 10 (2.3) | 0.014 |
| Chronic kidney disease | 22 (18.9) | 41 (9.5) | 0.005 |
| Immunocompromised status | 46 (39.7) | 78 (18.1) | < 0.001 |
| HIV | 2 (1.7) | 9 (2.1) | 1.000 |
| Long-term corticosteroids | 16 (13.8) | 41 (9.5) | 0.183 |
| Solid organ transplantation | 3 (2.6) | 12 (2.8) | 1.000 |
| Immunosuppressive drugs | 23 (19.8) | 58 (13.5) | 0.088 |
| Splenectomy | 0 (0) | 4 (0.9) | 0.583 |
| Current active neoplasia | 26 (22.4) | 25 (5.8) | < 0.001 |
| Recent chemotherapy | 12 (10.3) | 15 (3.5) | 0.003 |
| **Drug exposure during the 14 days before ICU admission** | | | |
| ACEi or ARB | 40 (34.5) | 133 (30.9) | 0.466 |
| NSAIDs | 1 (0.9) | 24 (5.6) | 0.031 |
| **Severity during the first day in ICU** | | | |
| SOFA | 9.6 ± 3.7 | 7.5 ± 3.3 | < 0.001 |
| SAPS II | 59.6 ± 19.4 | 47.4 ± 14.9 | < 0.001 |
| Invasive mechanical ventilation | 94 (81.8) | 345 (80.6) | 0.784 |
| PaO2/FiO2, mmHg | 112 ± 60 | 115 ± 56 | 0.647 |
| Shock | 83 (72.2) | 254 (59.4) | 0.012 |
| Glasgow coma scale | 13.4 ± 3.4 | 14.2 ± 2.2 | 0.033 |
| **Laboratory findings during the first day in ICU** | | | |
| Urea, mmol/L | 15.3 ± 10.4 | 12.5 ± 7.8 | 0.009 |
| Serum creatinine, µmol/L | 178.2 ± 180.4 | 158.5 ± 125.7 | 0.277 |
| CK, IU/L | 1365 ± 5164 | 4597 ± 20552 | 0.017 |
| Lactate, mmol/L | 3.2 ± 3.1 | 2.3 ± 1.5 | 0.003 |
| Leukocytes, G/L | 17.0 ± 37.3 | 15.1 ± 18.3 | 0.614 |
| CRP, mg/L | 70 ± 118 | 402 ± 155 | 0.055 |
| Proteinuria, g/L | 1.49 ± 1.47 | 1.44 ± 1.52 | 0.863 |
| Albuminuria, mg/L | 142 ± 87 | 388 ± 604 | 0.145 |
| Hematuria | 25 (44.6) | 103 (53.9) | 0.221 |
| Glycosuria | 8 (44.4) | 33 (43.4) | 0.937 |

Patients were compared according to the new onset AKI during the ICU stay, according to the 2012 Kidney Disease Improving Global Outcome (KDIGO).
Abbreviations: ACEi: angiotensin converting enzyme inhibitors, AKI: acute kidney injury, ARB: angiotensin II receptor blockers, COPD: chronic obstructive pulmonary disease, CK: creatine kinase, CRP: C-reactive protein, HIV: human immunodeficiency virus, ICU: intensive care unit, NSAIDs: non-steroidal anti-inflammatory drugs, $PaO_2/FiO_2$: ratio of partial pressure of arterial oxygen ($PaO_2$) to the fraction of inspired oxygen ($FIO_2$), SAPS II: Simplified Acute Physiology Score II, SOFA: Sequential Organ Failure Assessment.
Data are n (%) or mean ± standard deviation.

**Supplementary Table S6. Factors associated with D28 mortality, univariate analysis: in-ICU management, course, and outcomes**

| | **Dead at D28 (n = 116)** | **Alive at D28 (n = 430)** | **p** |
|---|---|---|---|
| **Anti-legionella antibiotics** | | | |
| Macrolide | 100 (89.3) | 396 (92.1) | 0.342 |
| Fluoroquinolone | 95 (84.8) | 405 (94.2) | 0.001 |
| Rifampicin | 13 (11.6) | 50 (11.6) | 0.995 |
| Dual antibiotic therapy | 88 (78.6) | 385 (89.5) | 0.002 |
| **Organ failures and supports during the ICU stay** | | | |
| ARDS Severity | | | 0.011 |
| Mild | 0 (0) | 9 (2.2) | |
| Moderate | 24 (21.1) | 132 (31.6) | |
| Severe | 88 (77.2) | 254 (59.4) | |
| Worst PaO2/FiO2 ratio, mmHg | 83 ± 40 | 95 ± 41 | 0.004 |
| Highest PEEP, $cmH_2O$ | 12.8 ± 3.3 | 12.9 ± 3.2 | 0.961 |
| Inhaled nitric oxide | 29 (25.0) | 79 (18.4) | 0.112 |
| ECMO | 19 (16.4) | 51 (11.9) | 0.196 |
| Shock | 110 (94.8) | 336 (78.1) | < 0.001 |
| Maximum norepinephrine, mg/h | 10.7 ± 9.1 | 3.6 ± 5.7 | < 0.001 |
| Corticosteroids | 34 (20.7) | 86 (20.0) | 0.870 |
| **ICU course and outcomes** | | | |
| Duration of mechanical ventilation, days | 7 [4;12] | 12 [7;20] | < 0.001 |
| Ventilator-free days at D28 | 0.7 ± 3.9 | 14.0 ± 8.4 | < 0.001 |
| Duration of ICU stay, days | 8 [4;14] | 17 [12;29] | < 0.001 |
| ICU-free days at D28 | 0.7 ± 3.7 | 9.7 ± 7.6 | < 0.001 |
| Hospital length of stay, days | 11 [6;18] | 30 [21;46] | < 0.001 |

Patients were compared according to the new onset AKI during the ICU stay, according to the 2012 Kidney Disease Improving Global Outcome (KDIGO).
AKI: Acute Kidney Injury, ECMO: Extra-Corporeal Membrane Oxygenation, ICU: intensive care unit, $PaO_2/FiO_2$: ratio of partial pressure of arterial oxygen ($PaO_2$) to the fraction of inspired oxygen ($FIO_2$), PEEP: Positive end-expiratory pressure.
Data are n (%) or mean ± standard deviation, except for time-dependent variables, which are expressed as median [interquartile range].

**Supplementary Table S7. Factors associated with D28 mortality, univariate analysis: kidney impairment**

| | Dead at D28 (n = 116) | Alive at D28 (n = 430) | p |
|---|---|---|---|
| Severity of AKI in ICU | | | < 0.001 |
| KDIGO 1 | 13 (11.2) | 94 (21.9) | |
| KDIGO 2 | 9 (17.8) | 47 (10.9) | |
| KDIGO 3 | 79 (68.1) | 164 (38.3) | |
| Need for RRT | 70 (60.3) | 130 (30.2) | <0.001 |
| Peak serum creatinine, µmol/L | 274.8 ± 199.9 | 238.8 ± 199.1 | 0.092 |
| Peak urea, mmol/L | 27.7 ± 14.8 | 22.9 ± 13.9 | 0.002 |
| Peak CK, IU/L | 5269 ± 28604 | 5501 ± 21144 | 0.947 |
| CK > 5000 IU/L | 8 (10.3) | 53 (16.9) | 0.149 |
| Peak LDH, U/L | 5967 ± 20741 | 1090 ± 2078 | 0.063 |

Patients were compared according to the new onset AKI during the ICU stay, according to the 2012 Kidney Disease Improving Global Outcome (KDIGO).
AKI: Acute Kidney Injury, CK: creatine kinase, ICU: intensive care unit, LDH, lactate dehydrogenase, RRT: renal replacement therapy
Data are n (%) or mean ± standard deviation, except for time-dependent variables, which are expressed as median [interquartile range].

**Supplementary Table S8: Sensitivity analysis: Multivariate analysis of variables associated with new-onset AKI and need for renal replacement therapy additionally adjusted for year of ICU admission**

| Risk factors | AKI | | Need for RRT | |
|---|---|---|---|---|
| | aOR (95%CI) | p | aOR (95%CI) | p |
| Age (years) | 1.00 [0.98 ; 1.03]* | 0.789 | 0.98 [0.96 ; 0.999]* | 0.039 |
| SAPS-II at D1 | 1.06 [1.04 ; 1.08]* | < 0.001 | 1.06 [1.04 ; 1.07]* | < 0.001 |
| Hypertension | 0.90 [0.51 ; 1.59] | 0.719 | 0.88 [0.52 ; 1.49] | 0.637 |
| Congestive heart failure | 4.05 [1.15 ; 14.30] | 0.012 | 1.17 [0.53 ; 2.57] | 0.702 |
| Chronic kidney disease | 7.07 [1.56 ; 31.12] | 0.011 | 7.76 [3.41 ; 17.65] | < 0.001 |
| Rhabdomyolysis (CK > 5000 IU/L) | 4.18 [1.55 ; 10.56] | 0.002 | 4.09 [2.18 ; 7.67] | < 0.001 |
| Year of ICU admission | 0.93 [0.85-1.01]* | 0.096 | 0.98 [0.91-1.06]* | 0.679 |

* For a one-unit increase.
aOR: adjusted odds ratio, AKI: Acute Kidney Injury, CK: Creatine kinase, ECMO: Extra-corporeal membrane oxygenation, ICU: Intensive Care Unit, RRT: Renal Replacement Therapy, SAPS-II: Simplified Acute Physiology Score II

## STROBE Statement — Checklist of items that should be included in reports of cohort studies

| | Item No | Recommendation | Page No |
|---|---|---|---|
| **Title and abstract** | 1 | (*a*) Indicate the study's design with a commonly used term in the title or the abstract | 1-3 |
| | | (*b*) Provide in the abstract an informative and balanced summary of what was done and what was found | 3 |
| **Introduction** | | | |
| Background/rationale | 2 | Explain the scientific background and rationale for the investigation being reported | 4 |
| Objectives | 3 | State specific objectives, including any prespecified hypotheses | 4-5 |
| **Methods** | | | |
| Study design | 4 | Present key elements of study design early in the paper | 5 |
| Setting | 5 | Describe the setting, locations, and relevant dates, including periods of recruitment, exposure, follow-up, and data collection | 5 |
| Participants | 6 | (*a*) Give the eligibility criteria, and the sources and methods of selection of participants. Describe methods of follow-up | 5 |
| | | (*b*) For matched studies, give matching criteria and number of exposed and unexposed | N/A |
| Variables | 7 | Clearly define all outcomes, exposures, predictors, potential confounders, and effect modifiers. Give diagnostic criteria, if applicable | 5 + Suppl methods |
| Data sources/ measurement | 8* | For each variable of interest, give sources of data and details of methods of assessment (measurement). Describe comparability of assessment methods if there is more than one group | 5 |
| Bias | 9 | Describe any efforts to address potential sources of bias | 5 |
| Study size | 10 | Explain how the study size was arrived at | 5 |
| Quantitative variables | 11 | Explain how quantitative variables were handled in the analyses. If applicable, describe which groupings were chosen and why | 6 |
| Statistical methods | 12 | (*a*) Describe all statistical methods, including those used to control for confounding | 6 |
| | | (*b*) Describe any methods used to examine subgroups and interactions | 6 |
| | | (*c*) Explain how missing data were addressed | 6 |
| | | (*d*) If applicable, explain how loss to follow-up was addressed | 6 |
| | | (*e*) Describe any sensitivity analyses | 6 |
| **Results** | | | |
| Participants | 13* | (a) Report numbers of individuals at each stage of study—eg numbers potentially eligible, examined for eligibility, confirmed eligible, included in the study, completing follow-up, and analysed | 6 |
| | | (b) Give reasons for non-participation at each stage | N/A |
| | | (c) Consider use of a flow diagram | - |
| Descriptive data | 14* | (a) Give characteristics of study participants (eg demographic, clinical, social) and information on exposures and potential confounders | 6-7 |
| | | (b) Indicate number of participants with missing data for each variable of interest | |
| | | (c) Summarise follow-up time (eg, average and total amount) | 7-9 |
| Outcome data | 15* | Report numbers of outcome events or summary measures over time | 7-9 |

| | | | |
|---|---|---|---|
| Main results | 16 | (*a*) Give unadjusted estimates and, if applicable, confounder-adjusted estimates and their precision (eg, 95% confidence interval). Make clear which confounders were adjusted for and why they were included<br>(*b*) Report category boundaries when continuous variables were categorized<br>(*c*) If relevant, consider translating estimates of relative risk into absolute risk for a meaningful time period | 7-9<br><br><br>N/A |
| Other analyses | 17 | Report other analyses done—eg analyses of subgroups and interactions, and sensitivity analyses | 8-9 |
| **Discussion** | | | |
| Key results | 18 | Summarise key results with reference to study objectives | 9-10 |
| Limitations | 19 | Discuss limitations of the study, taking into account sources of potential bias or imprecision. Discuss both direction and magnitude of any potential bias | 11 |
| Interpretation | 20 | Give a cautious overall interpretation of results considering objectives, limitations, multiplicity of analyses, results from similar studies, and other relevant evidence | 9-11 |
| Generalisability | 21 | Discuss the generalisability (external validity) of the study results | 11 |
| **Other information** | | | |
| Funding | 22 | Give the source of funding and the role of the funders for the present study and, if applicable, for the original study on which the present article is based | 21 |

*Give information separately for exposed and unexposed groups.

**Note:** An Explanation and Elaboration article discusses each checklist item and gives methodological background and published examples of transparent reporting. The STROBE checklist is best used in conjunction with this article (freely available on the Web sites of PLoS Medicine at http://www.plosmedicine.org/, Annals of Internal Medicine at http://www.annals.org/, and Epidemiology at http://www.epidem.com/). Information on the STROBE Initiative is available at http://www.strobe-statement.org.
